# The organizational dynamics enabling patient portal impacts upon organizational performance and patient health: a qualitative study of Kaiser Permanente

**DOI:** 10.1186/s12913-015-1208-2

**Published:** 2015-12-16

**Authors:** Terese Otte-Trojel, Thomas G. Rundall, Antoinette de Bont, Joris van de Klundert, Mary E. Reed

**Affiliations:** Institute of Health Policy & Management, Erasmus University Rotterdam, Rotterdam, The Netherlands; School of Public Health, University of California, Berkeley, Berkeley, CA USA; Kaiser Permanente Division of Research, Oakland, CA USA; NNIT, Public and Healthcare Advisory, Østmarken, 3A, 2800 Soeborg Denmark

**Keywords:** Patient portals, Personal health records, Kaiser Permanente, kp.org, Care delivery

## Abstract

**Background:**

Patient portals may lead to enhanced disease management, health plan retention, changes in channel utilization, and lower environmental waste. However, despite growing research on patient portals and their effects, our understanding of the organizational dynamics that explain how effects come about is limited.

**Methods:**

This paper uses qualitative methods to advance our understanding of the organizational dynamics that influence the impact of a patient portal on organizational performance and patient health. The study setting is Kaiser Permanente, the world’s largest not-for-profit integrated delivery system, which has been using a portal for over ten years. We interviewed eighteen physician leaders and executives particularly knowledgeable about the portal to learn about how they believe the patient portal works and what organizational factors affect its workings. Our analytical framework centered on two research questions. (1) How does the patient portal impact care delivery to produce the documented effects?; and (2) What are the important organizational factors that influence the patient portal’s development?

**Results:**

We identify five ways in which the patient portal may impact care delivery to produce reported effects. First, the portal’s ability to ease access to services improves some patients’ satisfaction as well as changes the way patients seek care. Second, the transparency and activation of information enable some patients to better manage their care. Third, care management may also be improved through augmented patient-physician interaction. This augmented interaction may also increase the ‘stickiness’ of some patients to their providers. Forth, a similar effect may be triggered by a closer connection between Kaiser Permanente and patients, which may reduce the likelihood that patients will switch health plans. Finally, the portal may induce efficiencies in physician workflow and administrative tasks, stimulating certain operational savings and deeper involvement of patients in medical decisions. Moreover, our analysis illuminated seven organizational factors of particular importance to the portal’s development - and thereby ability to impact care delivery: alignment with financial incentives, synergy with existing IT infrastructure and operations, physician-led governance, inclusive decision making and knowledge sharing, regional flexibility to implementation, continuous innovation, and emphasis on patient-centered design.

**Conclusions:**

These findings show how organizational dynamics enable the patient portal to affect care delivery by summoning organization-wide support for and use of a portal that meets patient needs.

## Background

Patient portals are secure websites that give health care consumers (here referred to as patients) access to personalized health records and typically include capabilities such as secure emailing with physicians, appointment scheduling, and educational programs [1]. Patient portals are different from personal health records (PHRs). The main difference between the two is ownership: whereas a patient portal is owned by the organization that provides it, patients typically own their PHRs [1].

Scholars suggest that patient portals can improve organizational performance, for example by decreasing the need for in-person visits (some of which may be substituted by secure emails), by letting patients manage more activities online (such as through online appointment scheduling and prescription refill), and by reducing the need for paper printouts and postage (enabled by online transmission of test results and care plans) [2, 3]. Relatively few studies, however, have documented such effects of patient portals on organizational performance [4, 5] and studies that have examined effects on healthcare utilization show mixed results [6].

Portals may also positively influence patient health by enabling and stimulating patients to manage and monitor their care [2], something that may be of particular value to patients with chronic diseases [7]. Numerous research studies have been conducted to assess the effects of patient portals (and similar EHR-linked online services) on patient outcomes, and systematic reviews of this rapidly growing literature have been published [4, 8–11]. Several studies found the use of patient portals to be correlated with better chronic disease management, expressed in terms of outcome indicators such as blood pressure and hemoglobin levels (see e.g., [12–14]). The effects are especially significant for patients with chronic diseases and when coupled to case management [4]. Also, several studies indicate that use of the portal is positively associated with patient satisfaction (e.g., [15, 16]). Yet, the strength of these correlations varies across studies and some studies did not find statistically significant associations between portal use and patient outcomes [11].

Despite the potential of patient portals to improve patient health and organizational performance, the inconsistent evidence reported thus far testifies to how difficult it is to realize and measure this potential. The variety of patient populations, portal functionalities, and contexts in which patient portals are implemented may explain some of the variation in results [17–19]. Also, the implementation of patient portals is a complex intervention, and the dynamics associated with such a change within the implementing organizations are likely to play an important role as well. Yet, within the current evidence base, the organizational factors influencing the impact of patient portals on patient health and organizational performance have received little attention. Published studies that document patient portal effects rarely include descriptions of the organizational dynamics that enable these effects [6]. Further, in a recent systematic review of the literature on developing patient portals, the authors observed a tendency for patient portal research reports to include information from the perspective of patients and providers, while efforts to understand the organizational dynamics from the lens of the clinical and managerial leaders that develop and implement a patient portal are sparse [20].

This study explicitly aims to advance understanding of the organizational dynamics that influence the impact of patient portals on organizational performance and patient health from the perspective of relevant organizational leaders. To this purpose, we conducted a qualitative study at Kaiser Permanente (KP), an organization for which some research on the effects of the patient portal on organizational performance and patient health has already been reported, thus giving us the opportunity to identify the organizational dynamics that influenced the development of a portal with organizational and patient outcomes. KP is a prepaid integrated delivery system operating on a global budget [21]. It consists of the Kaiser Foundation Health Plan & Hospitals and Medical Groups in each of the seven regions in which KP operates. The Medical Groups provide care to the 9.5 million members that are insured through the KP Health Plan [22].

KP’s patient portal, accessible through the website and through Apple and Android mobile applications, has evolved over the last 15 years. The portal is integrated with KP HealthConnect, a system-wide electronic health record (EHR) that was fully implemented across KP in 2010 [23]. Both the EHR and the patient portal are developed by the vendor, Epic Systems. In its current form, the portal gives patients access to parts of their medical records, encyclopedias and self-management programs. Also, it facilitates interaction between patients and physicians via secure email and, in some regions, video consult. Further, the portal offers transactional components including appointment scheduling, prescription refill, and insurance management tools [24]. With 4.4 million registered members as of October 2013, sending 14 million secure emails to 15,000 physicians per year, the KP portal is the most widely used privately owned patient portal in the world [25]. Due to its long running time, extensive patient use, and documented effects, KP’s portal makes an ideal case for our study. We now briefly summarize the reported effects of the portal on patient health and organizational performance.

Several research studies have reported beneficial effects of KP’s portal, including better blood pressure control, scores on the Healthcare Effectiveness Data and Information Set (HEDIS) [26–28] and use of preventive services for children [29]. Moreover, it has been found to positively influence patient experiences; one study demonstrated that users of the portal were more than two and a half times more likely to stay members of KP, likely due to enhanced satisfaction [30]. In addition, KP is one of the few systems that have reported variations in channel utilization after introduction of a patient portal [31–34]. Of these four studies, three found that the portal reduced utilization of telephone contacts, in-person doctor’s visits and hospitalization, while one documented that the portal led to increased use of these channels. (It may be that some of this inconsistency can be explained by the difficulty of accounting for pent-up demand prior to portal use or the fact that patient portal use may be a predictor for healthcare needs.) Finally, researchers at KP examined how the portal reduced the use of resources such as paper, postage, and gasoline, thus reducing environmental waste [35].

### Analytical framework

As indicated above, the KP portal has been shown to enable improvements in patient health and some measures of organizational performance. The contribution of this study is to advance our understanding of how these effects are realized.

Our analytical framework is the qualitative case study. Our approach is similar to the case study of KP completed by McCarthy et al. [22], which was designed to learn how the organizational context specific to KP supported care delivery to enhance the quality of in-patient care and ambulatory care. The case study approach allowed the authors to examine how KP scored on six organizational attributes believed to be of particular importance to achieving high-quality care delivery. These attributes included information continuity, care coordination, system accountability, peer review and teamwork, continuous innovation, and easy access to appropriate care. Further, the authors examined how these organizational attributes influenced care delivery at KP. For example, with regard to care coordination the authors found that the Northern California region used an extensive case management strategy for patients at risk of developing chronic diseases. By offering different combinations of primary and secondary care services to patients stratified by their level of risk, this strategy was successful in improving cholesterol screening, blood pressure control and appropriate receipt of medication while lowering mortality, smoking prevalence, and hospitalization rates for coronary heart disease and strokes. As an example of continuous innovation, KP has set up a Care Management Institute that brings together experts from all the regions to identify causes of variation and establish best practices for regional adoption. One result of the Institute’s efforts was an osteoporosis program in the Southern California region that led to significant reductions in the rate of hip fractures and needed treatments. As illustrated through these examples, the authors uncovered how organizational attributes of KP facilitated a number of care delivery processes to improve patient health and organizational performance.

Using a similar conceptual approach, this study will conduct a case study to examine organizational leaders’ understandings of the effects of the KP portal on patient care delivery and how the organizational context specific to KP influenced the patient portal’s development. Specifically, this study will assess how selected KP practitioners and executives who work closely with the portal believe that it impacts care delivery and how this led to improvements in patient health and organizational performance. By impacts on care delivery, we refer to how the portal alters care processes such as patients’ access to healthcare services and the relationships and interactions among patients and physicians. Subsequently, we will distinguish and illuminate organizational factors that have particularly influenced how the portal has been developed within the organization. We focus on organizational factors such as governance, leadership, vision, mission, core values and strategy, and organizational processes that have been shown to importantly influence organizational innovation and change in research or health care organizations [36]. We posit that these factors are of importance to the way a portal is being developed, and thus, its ability to produce effects. Based on these conceptual ideas, we used the following questions to frame our study: (1) How does the patient portal impact care delivery to produce the documented effects at KP? (2) What are the important organizational factors that influence the patient portal’s development?

Better understanding of how portals assist care delivery and what organizational factors influence the degree to which they do this is important to improving their performance. This understanding is especially important to health managers and practitioners developing patient portals as well as to researchers evaluating their effects.

## Methods

### Data collection

We conducted semi-structured interviews with a purposive sample of key organizational members who have been involved in the development, implementation and evaluation of the KP portal. Specifically, we interviewed 18 leaders, including physician leaders in the Northern California Permanente Medical Group and senior directors at KP’s National Program Office, between April and June 2014. Our respondents represented key divisions at KP working with the portal, including information technology, strategy, marketing, policy, and analytics, as well as physician leaders such as medical directors. By asking respondents to refer us to colleagues they knew to be particularly knowledgeable about the portal, we used the snowball technique to identify our purposive sample of respondents [37]. Each respondent confirmed that he or she directly worked on the development or implementation of the KP portal or was otherwise knowledgeable about the portal. We continued this sampling process until we reached saturation; that is, until we concluded that little new information came out of the interviews and no new candidate respondents were mentioned as imperative to our investigation [38]. Except for one senior director, all the persons we contacted agreed to participate in our study.

Based on formative research and discussion among the study team [6, 39], we developed an interview guide intended to elicit information relevant to answering our two research questions. Yet, to not let our questions direct or restrict the interviews, we took an open and exploratory approach, including a broad range of topics in our interview guide. We adjusted the guide to each respondent; however for all respondents the questions explored the following topics: organizational motivation for the patient portal; development and implementation of the portal; organizational processes to develop, maintain and operate the portal; governance, decision-making and funding of the portal; use of the patient portal; and the effects of the portal on patient health and organizational performance. To ensure we did not miss relevant information, we concluded each interview by asking if the respondent could think of topics relevant to the portal beyond those covered in the interview. One member (TOT), and in four cases two members (TOT and TR) of the study team conducted the interviews. The interviews were done by phone and lasted between 45 and 60 min. With permission of the respondents, the interviews were recorded and transcribed. Respondents were promised anonymity and all identifiers in our reporting of the interviews have been removed. Copies of the standard interview guide may be obtained from the corresponding author. The research was approved by the Kaiser Permanente Institutional Review Board.

### Data analysis

Following principles of content analysis, we coded the interview transcripts according to our two primary research questions [40]. Thus, one category contained information about the portal’s impact on care delivery; and one about the organizational factors that influence the development of the portal. Within each of these categories, we subsequently coded the data under headings and subheadings that emerged during the analysis, thereby identifying recurring themes [41]. One member of the study team (TOT) first coded the data. A second member of the study team (TR) reviewed each coded text phrase, reading the phrase in the original text and judged whether the code was applied correctly. This reliability check concluded a 95 % agreement rate. Where there were variations in coding decisions, the codes were discussed until agreement was reached [42]. We performed a member check on our qualitative analysis and interpretation of the responses to our questions by sharing and discussing our findings with two of our respondents, thereby seeking to verify the content and the weight we placed on various topics.

## Results

We report the findings of our content analysis by addressing our two research questions. Below, we identify the themes that emerged from our analysis and use relevant quotes from the interviews to provide specific examples within each theme.

### How does the patient portal impact care delivery to produce the documented effects at KP?

As indicated in the background, previous research found that KP’s patient portal has improved clinical process measures and member retention and elicited some operational savings and changes in channel utilization. Our analysis of the interview data uncovered five ways our respondents believed that the patient portal affected care delivery to produce these outcomes. As we describe in more detail below, respondents reported that the portal generates the outcomes by improving ease of access, transparency of information, patient-physician interaction, connection with members, and operational efficiency. While the first ways describe impacts at the patient level, the latter are primarily centered on the organization. The chosen listing order thus reflects a focus from the patient level to the organizational level.

#### Ease of access

Our respondents emphasized the ability of the portal to ease patients’ access to the system. For example, the transactional capabilities simplify the managing of care, since patients do not have to hold on the phone, can perform transactions at their convenience, and can avoid taking time off work to seek medical care (if the issue can be handled per email or e-consult). Especially the convenience of asynchronous communication was emphasized in the interviews; patients can contact their physicians directly with questions when they arise, and outside of regular business hours. As one respondent said, *“I think that the portal does a lot in terms of access by giving patients more direct access to their physicians and reducing the time they have to take off work.”* Our respondents assumed that this improved ease of access enhanced patient satisfaction, and by extension, patients’ proclivity to remain a member of the delivery system. According to our respondents, for many patients, having new and more convenient ways to access their physicians can also impact channel utilization. Giving patients easy, direct access to their team of physicians via the portal may reduce the perceived need for in-person visits, as some inquiries and transactions can be handled via email. Yet, easy-to-use portal communications may also lower the threshold for seeking contact with physicians so that secure emailing is rather used in addition to these other channels.

#### Transparency of information

Prevalent among the respondents was the notion that by giving patients an overview of their medical records, care plans and lab results the portal educates patients about their health. This transparency of information to patients was thought to make them better able to participate in their care as informed partners. Also, up-to-date information, such as lab results, might activate patients (particularly those with a certain level of medical literacy) to make positive strides to affect their health or serve as reminders to improve their health habits. Further, follow-up emails reinforce the message given in the physician’s office, which can improve patient adherence and understanding. One respondent expressed: *“You’re helping them get the information they need to improve their health. You’re giving them greater access to information that can help them manage their chronic conditions, for example.”* According to our respondents, these are mechanisms by which the portal improves quality scores (HEDIS), clinical process measures, and use of preventive services.

#### Patient-physician interaction

The portal permits physicians to send follow-up messages, reminders about screenings and tests, and other information to make their patients better informed. A respondent expressed that *“they [physicians] can for example see when someone is due for their cholesterol testing and they can send a note reminding the patient and placing an order in the system”.* This reinforcement of messages enabled by secure email may enhance some patients’ adherence to treatment, medications, and lifestyle changes, thereby aiding health promotion or disease prevention or management. One respondent explained that *“Its reinforcing a message, for example that “you need to take, stay or get back on this particular drug”. I think it gives an opportunity to engage patients in getting that message and maybe adhering a bit better to some of the protocols”.* Further, some respondents expressed that electronic communication can help physicians notice more issues than if they relied solely on face-to-face interaction or phone conversations alone. For example, patients can use the portal to update their medications list, thereby assisting physicians in assuring medications are appropriate given the patient’s current medical condition. Through these pathways the portals may improve the quality of patient care. Moreover, several respondents noted that the continuity of contact facilitated by secure messaging improves the interaction between patients and their physicians and thereby *“augments the connection between patients and their providers”*. Also, since patients and physicians have access to the same information, use of the patient portal can help streamline discussions and improve the quality of communication. This positive influence on the quality of the physician-patient relationship may, according to several respondents, improve patients’ attachment to their physicians, thereby increasing the probability that they will remain members of the health plan.

#### Connection with patients

Another way by which the portal can enhance member retention is by helping KP strengthen its connection with its patients. Several respondents explained how the portal enables personalization of services and extension of services into patients’ lives. In the future, this may be reinforced by expanded patient portal capabilities that will allow patients to, for example, upload data from wellness devices onto the patient portal. Moreover, the portal functions as an additional medium for KP to communicate with and engage their patients. One respondent said that, *“Digital enables that constant stream of communication where the patients can pull and not just be the result of a push. It can drive engagement to a completely different level.”* More generally, the patient portal is believed to make a positive contribution to KP’s image.

#### Operational efficiency

Our respondents reported that the portal improves operations at KP in several ways, including administration and physician workflow. First, it allows for administrative efficiencies by reducing the need to call patients about test results, book appointments by phone, and to use paper and postage for communication with patients. As one respondent expressed: *“By creating your own appointments - as opposed to having to use up somebody’s time on the phone or using up a receptionist’s time to create an appointment - by doing self-service, patients can do it for themselves and do it more efficiently. We save paper, postage, because we don’t have to mail out the lab results. They can be viewed online. There are enormous efficiencies that come to Kaiser because of the fact that we’re online.”* Furthermore, some respondents referred to an internal (and unpublished) study that found that patients who book their own appointments through the portal have a lower no-show rate and speculated that the increased number of people that use this feature might reduce this waste. Moreover, the clarity and quality of the information that is inserted in the EHR (at least the portion that is transferred to the portal) tends to be clearer and more accurate since it has to be reported in a way that is understandable to patients. This may reduce inefficiencies stemming from errors or misinterpretation among healthcare professionals as well as between patients and their physicians. Second, the portal, and especially the secure emailing functionality, redefines physician workflow. The introduction of secure emailing clearly increased the volume of patient emails that physicians must respond to. However, the general expectation among our respondents was that this extra volume can be balanced by better-managed patient panels, thus reducing the overall patient demand for in-person visits and telephone calls. A respondent explained that: *“The hope is that they’ll [physicians] have more accountability for their panel. So the incentive would be that if they are able to better manage their panel through the portal, then maybe they can have more time during the day for email, because fewer patients need to come in, rather than just having the call center loading them up with more and more appointments.”* This, in turn *“really frees up the providers’ and physicians’ time to focus on more of the complex things”.* Further, physicians are able to respond to messages in an asynchronous fashion at their convenience and can avoid ‘playing phone tag’ with patients. Our respondents believe that the efficiencies described above result in cost savings, including reduced environmental waste, also reported by Turley et al. [35].

**Fig. 1:**
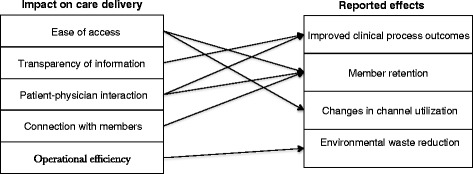
Associations between KP’s patient portal’s impacts on care delivery and reported effects

Figure 1 summarizes the proposed associations between the portal’s impacts on care delivery and documented effects. It should be noted that the arrows signify the associations that most often surfaced from the interview data, and do not mean to imply that other associations between care delivery impacts and patient portal effects do not exist. Also, our research approach does not support claims about how these associations may play out across different patient sub-populations, such as age or health status groups. Similar to the impacts, we list the effects of the patient portal from most to least patient-specific.

The purpose of this study was to understand how the KP portal has been able to affect operational performance and patient health. In establishing these associations, we have focused on the positive impacts of the portal on care delivery outlined in the introduction. Nevertheless, other implications of the portal on the care delivered to patients also surfaced in the interviews. These include risks that patients cannot cope with information online, depersonalization of care, and increased provider workload.

First, a few respondents described that for some patients, the increased transparency of information can cause anxiety, especially if they have to wait for a response from their physicians to interpret or cope with the information. *“The question is whether the transparency could cause anxiety to the patients. Seeing all that information, does that make patients more informed and active partners in their care, or does it make them feel more worried about a slight and insignificant abnormality”?* Second, another respondent pointed out that some patients are concerned that technology will distance them from their doctor, since it may replace some one-on-one interactions. *“There’s a risk that some members could feel distanced from their doctors if they never get to see them. If they feel that too much is being managed electronically or remotely. So we have to be careful about maintaining those relationships.”* Third, some respondents presented accounts that the introduction of secure email has led to a higher workload for some physicians due to the sheer volume of emails that came after the feature became available, and, as one respondent mentioned, some physicians have *“struggled with it and certainly with the feature that we provide where you can e-mail your doctor”.* These struggles may be associated with altered expectations about timeliness of response and the challenge for physicians of finding available time in their day to process the messages.

### What are the important organizational factors that influence the patient portal’s development?

Our analysis of the interview data coded under this question exposed seven factors that respondents claimed particularly influence patient portal. These factors include: (1) alignment with financial incentives, (2) synergy with technical and operational infrastructure, (3) physician-led governance, (4) inclusive decision making and knowledge exchange, (5) regional flexibility, (6) continuous innovation, and (7) patient-centered design.

#### Alignment with financial incentives

Many respondents pointed out that KP’s capitated reimbursement model (i.e., physicians are paid a set amount for taking care of a set amount of patients over a set period of time) has allowed for investments in consumer technology that lead to better patient care and substitution towards use of virtual services. As one respondent explained, *“The capitated model is part of why we were able to be so focused on it [the portal] in the first place. It is the right way to do things, it a cost-effective way to do things, and it provides quality and convenience to our members.” Furthermore, one respondent pointed out that, in contrast to fee-for-service models, “Kaiser doesn’t have to pay doctors on an e-mail-by-email basis. Kaiser is incenting the physician to take care of a panel of patients.”* Hence, physicians are believed to be more easily motivated to use the portal to better manage their patient panels and to be unaffected by how emailing is compensated relative to in-person visits.

#### Synergy with IT infrastructure and operations

The majority of respondents touched upon how the functionality of the patient portal is closely related to the technical and operational infrastructure it ties into. In terms of the technical infrastructure, since it is tethered to an organization-wide EHR, recorded data is constantly and automatically fed to the portal, giving patients up-to-date information from all their providers. In terms of the operational infrastructure, the patient portal creates synergies with existing case management efforts within KP by supporting better coordination and connecting the patients closer to their care teams. Ensuring such positive interactions between the patient portal and existing operational infrastructures is central. A respondent, who has been closely involved with KP’s information system development, noted that even previous to the portal’s inception there was a lot of groundwork done to *“better understand the synergies between what Kaiser was trying to accomplish and the emerging capabilities of technology.”*

#### Physician-led governance

Many respondents made the point that the portal has been developed and made possible through strong physician leadership. As one respondent said: “We’ve had some very bold, and brave, physician leaders who once […] understanding that the industry was changing and needed to change, said, *“this is part of what the medical group will now offer. This is part of how we will offer care.”* The CEOs of the KP Health Plan & Hospitals and the Medical Directors agreed that the Medical Groups should be in charge of setting the direction for KP’s consumer technology. According to several respondents, this entails both setting the strategic direction for the patient portal, and, on a more practical level, prioritizing the development of clinical features. This governance process takes place within the ‘Health Strategy & Governance Group’, with voting members drawn from each self-governing regional Medical Group. This governance group serves as a ‘clearinghouse’ for the regions by incorporating regional operational and clinical needs into the priorities that are set for the work done on the portal each year.

#### Inclusive decision-making and knowledge sharing

Although the respondents generally believed that there is alignment of priorities at a high level, some noted that organizational sub-units set priorities based on their own local needs. Staff in care delivery, for example, tend to be focused on portal-enabled clinical tools and may not see the same value in making financial functions available to patients as do administrative staff: One respondent said: *“I think that there are people in care delivery who are not much interested in using kp.org as a marketing tool and device, because they want to focus on care delivery.”* Several respondents expressed that this leads to some tension over specific priorities for a year. Hence, an important aspect for KP is to share knowledge among and obtain buy-in concerning the portal from various organizational members. To this end, KP makes use of experience design workshops, decision-making meetings, and a general effort to share best practices. Furthermore, collaborative decision-making where various groups come together, openly present their interests, and make decisions that benefit the overall strategy is a decision-making process that is widely used within KP to achieve directional alignment around core goals. This was elaborated by one respondent: *“It enables us to take the input from member experience and panel input in terms of what members want, the regional operations view of what regional operations want, the physician view of what physicians want, the nursing view of what nurses want, etc. It enables us to put all those different interests and objectives into the hopper and to try to solve for all of them together without bargaining or voting on each part individually*.” Many other comments referencing ‘engaging’, ‘we’, and ‘act without bargaining or voting’ conveyed shared values among organizational members that go beyond inclusive decision-making.

#### Regional flexibility

A recurring theme in the interviews was that the seven KP regions have different local priorities, resources, operating procedures, and even regulatory environments. Also, although KP has one EHR, most regions still use some legacy systems that were in place before the EHR was adopted. Therefore, some respondents explained how the national portal leadership work with each region to identify their needs and incorporate these into the design, implementation and rollout in each region. Acknowledging that implementing a new capability has differing implications for operations across the regions, the portal leadership uses a flexible implementation approach. Each new patient portal capability is made available to all regions, but depending on the individual region’s priorities and operational workflow issues, they may decide whether and how to ‘turn it on’. A respondent described this process: *“Everything that is on the portal is available to all regions if their regional operating procedures, or even their regulatory environment will allow them to have it. So, yes, everything is available, but there are regional differences and that’s just part of the nature of the beast, just because of where they operate in the US.”*

#### Continuous innovation

Several respondents commented that KP was an early EHR and patient portal adopter and has used the portal as a market differentiator. However, since many EHR and patient portal features have now become standard across health systems, KP recognizes the need to differentiate itself by further innovating on consumer technology. One respondent explained that: *“What we’re trying to do in terms of the marketplace, so more on the business side and for our customers, is to be able to keep offering rich and new experiences that we can market to those folks and set ourselves up as a differentiator in the marketplace. It has to be useful, helpful, and really great for the current membership. It also has to appeal to attracting, new customers”.* For example, KP is looking to companies in other industries, such as Apple and Amazon, to learn how those organizations manage their online presence and consumer experiences. In fact, continuous innovation is built into the development strategy for the patient portal; each year the group dedicated to developing the portal, in consultation with its IT counterpart, goes through a process of generating ideas and writing business cases for how to develop the portal in support of the overall strategy. As a result of this process, each year a number of projects are prioritized and receive funding.

#### Patient-centered design

KP uses a patient-centered approach focused on simplifying and enriching the patient experience. According to one respondent *“What I view as going to be key in driving engagement is a personalized, actionable, insightful experience. Even if you have all that data and features, but […] it doesn't match how consumers want to be able to access it […] it’s not going to drive that experience. We really need to think about how do we create that personalized experience and make it truly actionable”.* For instance, KP is currently focusing innovation on responsive design; building capabilities for all platforms (web, smartphones and tablets) to make the development of the patient portal more streamlined within KP and the user experience more consistent. The respondents involved in designing the portal explained how each new capability goes through extensive usability testing. Also, there is much communication between the developers and member services and advice call centers to ensure a constant cycle of validation of existing features. Moreover, KP actively promotes the portal to its patients and seeks to improve access. Upon registration, each new member is encouraged to sign up and KP monitors use and does outreach to non-users. Further, there are free classes to teach patients how to register and use the patient portal. To include more of its non-English speaking patient base, KP recently launched the portal in Spanish.

Again, we note that in line with our study aim, we have focused on enabling factors in our reporting of the key organizational factors that influence the portal. Nevertheless, some organizational factors that may negatively affect the portal also emerged in the interviews. For example, most respondents acknowledged that the size and complexity of KP, including the multi-state operations, pose challenges to any implementation project. Each region is exposed to different regulatory environments. Moreover, the organizational complexity either results in, or is a result of, frequent reorganizations with subsequent changes in the roles and responsibilities of divisions, groups, and people. Some respondents noted that these organizational changes complicate the establishment of working relationships, communication channels, and routines. Nevertheless, it was our perception that our respondents were so used to working under these conditions that they saw these issues as natural consequences of doing work within KP rather than as a barrier specific to the patient portal itself.

## Discussion

### Summary of findings

Through interviews with key organizational members, we have described how they perceive KP’s patient portal to be affecting care delivery to produce effects, and further, the organizational factors that have influenced the portal’s development. Below, we combine the findings from the content analysis of our interviews to see their interconnections more clearly, and where possible we assess the findings in the context of existing research in other care delivery organizations and within KP.

KP’s leaders believe that investing in the portal aligns with several of the organization’s operational goals, such as improving quality and access that, in turn, lead to increasing retention of better-managed patients with associated cost savings in the long term. These beliefs are consistent with existing research on patient portals (and similar EHR-linked online services) generally and in KP specifically [9, 30]. However, there is also evidence from many studies, including one done within KP, that there are significant disparities in the use of patient portals across socio-economic groups, and the increase in patient satisfaction and retention due to the use of a portal varies across sex, age, income, racial and ethnic groups and health literacy [4, 43]. While our respondents did not comment extensively on these disparities, they did refer to several initiatives within KP to support access for different demographic groups such as free classes on how to access and use the portal and translation of the portal.

Our respondents also believe that KP’s model of capitated payment of physicians provides incentives to physicians to use the portal to manage their patient panels. This belief is consistent with economic theory and the observation of many analysts that capitated physician payment motivates KP’s physicians to engage in practices that keep patients healthy while optimizing utilization [44].

Our respondents reported that the patient portal’s linkage to existing IT infrastructure, particularly the comprehensive EHR, powers its functionality and connectivity since patients are provided access to information from all their providers on one patient portal. This is consistent with the findings regarding patient satisfaction with portals in the systematic review reported by de Lusignan, et al. [9]. Respondents also noted that the use of the patient portal to support existing operations such as care coordination and case management makes its practical contribution to care delivery visible to physicians and other professionals working at the frontline of care. The portal’s alignment with organizational and physician incentives and programs to improve care delivery, and the visibility of that alignment, has allowed for large investments in and support of the use of the portal. Both aspects have been vital to developing and deploying a highly functional and comprehensive portal, a prerequisite for enabling its impact on care delivery.

The governance process that included ‘physician-led governance’, ‘inclusive decision-making and knowledge sharing’, and ‘regional flexibility’ has fostered a notion throughout KP that use of the patient portal is consistent with organizational and professional values. Our respondents reported that the governance process not only ensures that the portal meets clinical and operational needs, but also helps secure physician buy-in. The inclusive decision-making process that invites the opinion of various organizational members, along with efforts to share knowledge, facilitates a common understanding about the contributions of the portal to care delivery, such as bringing a better experience to the patient. To the extent that feedback from organizational members with varying interests is taken into account, the functions of the patient portal will better match the needs of these members. Further, the implementation strategy that enables each region to adopt patient portal features in a manner supportive of their priorities and operational realities fosters the belief that the portal is in line with clinical and operational needs pertinent to the individual regions. Our respondents believed that efforts to make the portal consistent with organizational and professional values make it more likely that the portal will be widely used and promoted by physicians and other staff across the organization. This participation is necessary to stimulate impacts on care delivery processes that rely on an active role of physicians to stimulate workflow and workload changes and to improve their interaction with patients and thereby the quality of care delivered. These observations are consistent with research in various related fields, including collaborative, participatory, and natural decision-making (e.g., [45]).

Finally, KP’s leaders believed that organizational factors concerning the portal’s development and design, specifically ‘continuous innovation’ and ‘patient-centered design’, contribute to the development of the patient portal in ways that meet patient needs and wishes. The aim of the continuous efforts to improve the capabilities of the portal is to match the potential of IT seen in other industries and to expand its meaningfulness to patients. Continuous innovation is closely related to the concept of continuous quality improvement (CQI), which is a well-established approach to improving quality of service and patient care in healthcare (and other) organizations. Numerous studies have demonstrated positive effects of CQI on organizational performance [46], which provides support for the validity of the beliefs held by our respondents. The patient-centered approach to designing capabilities strives to improve patients’ experience of navigating the health system and managing their care, an approach that is gaining acceptance in health care organizations and specifically in the design of personal health records [47, 48]. Through this constant improvement of capabilities combined with proactive outreach to non-users, KP hopes to make the portal play a progressively greater role in its service provision. Extensive patient use is essential to stimulating the portal’s impacts on patient care delivery, the ability of physicians to realize workflow and workload changes, and the ability of the organization to improve business operations as well as create a stronger connection with patients.

In sum, our respondents believed the dynamics between the organizational factors and the patient portal will trigger improvements in patient health and organizational performance at KP by enabling the embedding of comprehensive and functional patient portal services into care delivery throughout the organization. These beliefs are largely consistent with existing research on portals and related EHR-linked online services in other organizations as well as within KP.

### Transferability of findings

Being a large integrated delivery system, KP is uniquely different from other types of organizational settings such as individual hospitals and physician practices or networks of independent practices. In fact, the integrated setup is widely considered to be supportive of quality improvement and performance on a range of quality and efficiency measures [49–51]. In this paper, we have described key organizational dynamics pertinent to the integrated care delivery context of KP that our respondents believe support the patient portal. For example, the alignment between the patient portal and KP’s business case (e.g., the benefit of more loyal and better-managed patients) enables value realization [52]. The alignment has permitted KP to invest in the patient portal on the premise that it increases quality at a reasonable cost [53]. Yet, this may be different for other organizations, especially those operating with fee-for-service payment models [21]. For such organizations, revenues are proportional to patient visits or treatments (at least under models that do not, or only partly, reimburse e-visits.). Thus, offering a patient portal that may substitute for in-person visits and contribute to fewer patient health needs, contradicts this incentive structure [54]. Also, the high connectivity made possible through the portal’s linkage with a comprehensive EHR that covers all 9.5 million KP members and their providers amplifies its functionality, since it gives patients a ‘one stop shop’ and supports care coordination [55]. Achieving a similar connectivity for patient portals provided by independent practices will depend on integrating information from (typically) distinct information systems [56]. This requires not only technical, but also semantic interoperability to enable the meaningful use of information across practices [57]. Similarly, for each of the organizational factors we have identified, their (direct) transferability from KP to other organizations is unclear.

Nevertheless, even though information about KP’s patient portal may not be directly transferable to other types of organizations, the detailed information we provide allows practitioners and managers in any type of organization to distill and translate learning that could apply to their organizations. With this study of one particular portal in one particular system context, we have only started to develop the knowledge base on organizational dynamics influencing patient portals. We welcome research that further explores the impact of patient portals, and especially in more fragmented care delivery settings. Considering the increasing development of patient portals that is currently taking place in various organizational settings [2], research into what works, what does not work, and why, is relevant and timely.

### Study limitations

We interviewed only a fixed number of informants. While they largely represented the organizational groups responsible for the portal, they did not represent all of the leadership involved in the patient portal. Moreover, there is a risk that our respondents, due to loyalty to their organization and colleagues, have limited their responses to convey only positive aspects of KP and the patient portal. However, it is our impression that our respondents presented a balanced story, and appreciated the opportunity to reflect on aspects that work well and less well. It is also our sense that the promised anonymity created a comfortable forum for the respondents to share their knowledge with us.

It would have been interesting to complement the perspectives of the organizational members with those of patients. Of particular interest would be an exploration of how and why different patient populations reap benefits from care delivery assisted by a portal, thereby contributing to explaining the variance in effects across these populations. Nevertheless, the aim of this particular research was to describe the workings of a patient portal from the viewpoint of the organization that provides it.

Further, we have pointed to positive as well as negative impacts of the patient portal on patient care and provider workflow. For example, while enhanced use of emails may make provider workflow more flexible and allow for better patient panel management, it may also result in an enhanced workload for some providers. A limitation of our study is uncertainty about the relative contributions of reports regarding such impacts and the contexts that might drive them. Future research is needed to quantify these contributions and understand their context dependencies.

## Conclusions

Using semi-structured interviews, we addressed two research questions: (1) How does the patient portal impact care delivery to produce the documented effects at KP? and (2) What are the important organizational factors that influence the patient portal’s development, and thus, its impact on care delivery? To answer the first question, we identified ways our respondents believed that the portal affected care delivery to produce reported effects including enhanced disease management, health plan retention, changes in channel utilization, and lower environmental waste. We proposed that the portal’s ability to ease access to services improves some patients’ satisfaction, and thus health plan retention as well as stimulates changes in the way patients use channels to seek care. Further, the transparency and activation of information makes some patients better able to manage their care. Care management may also be improved through enhanced patient-physician interaction. Improved relationships between patients and providers made possible through enhanced interaction may also increase the ‘stickiness’ of some patients to their providers, reducing the likelihood that they will switch health plans. A similar effect may be triggered by the portal’s ability to improve the connection between KP and its patients. Finally, the portal may induce efficiencies in physician workflow and administrative tasks, leading to operational savings such as reduced environmental waste. While these impacts are generally perceived to be positive, we have made note of their bi-directional nature: depending on the conditions under which the patient portal is being used and the characteristics of the professionals and patients that use them, they may have perceived negative impacts. Among the negative impacts we have highlighted are enhanced or disrupted workflow or reduced personal contact between professionals and patients.

In answering our second research question, our analysis surfaced seven organizational factors of particular influence on the patient portal’s development. These factors include the portal’s alignment with financial incentives, synergy with existing IT infrastructure and operations, physician-led governance, inclusive decision making and knowledge sharing, regional flexibility to implementation, continuous innovation, and finally, KP’s emphasis on patient-centered design. These findings provide insights into how the organization enables the patient portal to affect care delivery by summoning organization-wide support for and use of a portal that meets patient needs. Yet, since these findings originate from a unique integrated delivery system, direct transferability of these findings to other types of care delivery systems may be limited.

## Abbreviations

PHR: Personal health record
KP: Kaiser Permanente
EHR: Electronic health record
HEDIS: Healthcare effectiveness data and information set
CQI: Continuous quality improvement
